# Declines in plasma erythritol levels and improvements in insulin sensitivity and glucose metabolism: POUNDS lost trial

**DOI:** 10.1038/s41366-026-02152-w

**Published:** 2026-07-21

**Authors:** Yoriko Heianza, Jennifer C. Rood, Catherine M. Champagne, Frank B. Hu, George A. Bray, Frank M. Sacks, Lu Qi

**Affiliations:** 1 Department of Epidemiology, Celia Scott Weatherhead School of Public Health and Tropical Medicine, Tulane University, New Orleans, LA USA; 2 Department of Nutrition, Harvard T.H. Chan School of Public Health, Boston, MA USA; 3 Pennington Biomedical Research Center, Louisiana State University, Baton Rouge, LA USA; 4 Department of Epidemiology, Harvard T.H. Chan School of Public Health, Boston, MA USA

**Keywords:** Obesity, Metabolic syndrome, Obesity

## Abstract

**Background:**

Erythritol is a non-nutritive sweetener that is also endogenously produced from glucose. We investigated whether declines in plasma erythritol and other sugar alcohols (polyols) in response to weight-loss dietary interventions were significantly associated with long-term improvements in glucose metabolism and insulin sensitivity.

**Methods:**

Plasma erythritol, mannitol/sorbitol, and arabitol/xylitol were repeatedly measured among adults with overweight or obesity who participated in the POUNDS Lost trial (*n* = 802 at baseline and *n* = 664 at 6 months). We analyzed associations of changes in erythritol, mannitol/sorbitol, and arabitol/xylitol from baseline to 6 months with changes in homeostasis model assessment of insulin resistance (HOMA-IR), fasting insulin, fasting glucose, and HbA1c at 6 months and 2 years of intervention.

**Results:**

Plasma erythritol levels were positively related to HOMA-IR, fasting insulin, and glycemic markers at baseline. Greater declines in plasma erythritol in response to the interventions were associated with greater reductions in HOMA-IR (*p* < 0.001) and fasting insulin (*p* < 0.001) at 6 months. Similar associations were found for changes in arabitol/xylitol, but not for mannitol/sorbitol. Every 1 SD reduction in plasma erythritol during the initial 6-month period was related to long-term (2-year) decreases in log-transformed HOMA-IR (β [SE] –0.07 [0.02], *p* = 0.008) and glycemic markers (β –1.0 [0.44] mg/dL, *p* = 0.025 for fasting glucose; β –0.05% [0.02%], *p* = 0.019 for HbA1c).

**Conclusion:**

Declines in plasma erythritol may be related to long-term improvements in insulin sensitivity and glycemic status during weight-loss dietary interventions in adults with overweight or obesity.

## Introduction

Sugar alcohols (polyols) are natural sweeteners that are considered “generally recognized as safe” (GRAS) substances by the U.S. Food and Drug Administration (FDA) and are approved for use as food additives in various products. Recent studies have demonstrated that elevated plasma levels of polyols, such as erythritol, sorbitol/mannitol, and xylitol, are associated with increased risks of central adiposity [1, 2], type 2 diabetes (T2D) [3], and other obesity-related chronic metabolic diseases [2, 4–7].

Among sugar alcohols, erythritol production by the food industry and its consumption by individuals are expected to increase [8, 9]. Erythritol is a natural sweetener that is also endogenously produced from glucose via the pentose-phosphate pathway [1, 10]. Therefore, circulating levels of erythritol are regulated by both endogenous production and by exogenous intake [1, 10, 11], such as foods naturally containing erythritol and processed foods containing artificial erythritol. Erythritol ingestion and other dietary factors (such as sucrose) may lead to prolonged increases in plasma erythritol concentrations above baseline/endogenous concentrations [4, 12, 13]. Elevated erythritol levels have been associated with microvascular complications of diabetes [14] and thrombosis risk [4]. On the other hand, prior studies reported that polyols did not affect abdominal fat or acute or short-term impairments in glycemic responses [15, 16]. Whether temporal changes in circulating erythritol levels, particularly in response to weight-loss interventions, are associated with long-term changes in insulin sensitivity and glucose metabolism remains unknown.

In the present study, we investigated whether 6-month changes in plasma erythritol and other polyols (including sorbitol/mannitol and arabitol/xylitol) in response to weight-loss diets were associated with improvements in insulin sensitivity and glucose metabolism over 2 years in adults with overweight or obesity participating in the POUNDS Lost trial.

## Subjects and methods

### Study design, setting, and participants

This study is an ancillary analysis of data from the POUNDS Lost trial (NCT00072995), which was conducted from October 2004 through December 2007 at two sites: Harvard T.H. Chan School of Public Health and Brigham and Women’s Hospital in Boston, MA, and the Pennington Biomedical Research Center of Louisiana State University System in Baton Rouge, LA [17]. The study was approved by the human subjects committee at each institution and by a data and safety monitoring board appointed by the National Heart, Lung, and Blood Institute. All participants gave written informed consent.

In the POUNDS Lost trial, 811 adults who were overweight or obese were randomly assigned to 1 of 4 energy-reduced diets with different macronutrient intake goals: (1) low-fat and average-protein: (20% fat, 15% protein, 65% carbohydrate), (2) low-fat and high-protein: (20% fat, 25% protein, 55% carbohydrate), (3) high-fat and average-protein: (40% fat, 15% protein, 45% carbohydrate), and (4) high-fat and high-protein: (40% fat, 25% protein, 35% carbohydrate). Therefore, carbohydrate content ranged from 35 to 65% of total energy (35–65% E) across the four diet groups, with 10%E increments between groups. The trial did not include specific advice on modifying intake of non-nutritive artificial sweeteners, so participants self-selected their use of natural and artificial sweeteners during the interventions. Blinding was maintained by naming each diet with colors and using similar foods across diets, and investigators who measured outcomes were unaware of participants’ diet assignments. The goal for physical activity was 90 min of moderate exercise per week, and the original trial reported that energy intake and physical activity were similar across the diet groups [17]. Smoking status was not a focus of the trial; however, only a small proportion of participants were current smokers (4%) among the 811 participants. Major exclusion criteria included diabetes or unstable cardiovascular disease, elevated urinary albumin excretion, use of medications that affect body weight, and insufficient motivation [17]. The follow-up rate was high, and 80% of participants completed the trial [17].

In the original trial, 811, 681, and 575 participants provided fasting blood samples at baseline, 6 months, and 2 years, respectively, and were eligible for the global metabolomics analysis. We analyzed metabolites in 2012 plasma samples, including 805 samples at baseline, 673 at 6 months, and 534 at 2 years, based on the availability of stored samples. After excluding samples measured only at 6 months (*n* = 3) or 2 years (*n* = 1), we had baseline data for 805 individuals, of whom 670 and 533 had repeated global metabolomics data at 6 months and 2 years, respectively. Of the 805 participants, we excluded baseline log-transformed erythritol values exceeding ± 5 SDs from the mean (*n* = 3), leaving 802 participants eligible for the analysis of baseline associations. The same QC was applied to other metabolite levels at baseline and 6 months; 664 had data on changes in erythritol from baseline to 6 months. Participants with missing data on outcome measures (HbA1c, fasting insulin, or the homeostasis model assessment-of-insulin resistance [HOMA-IR]) at baseline or at a follow-up point were excluded when performing each analysis.

### Measurements of plasma sugar alcohols and other metabolites

Fasting blood samples were collected at baseline, 6 months, and 2 years during the intervention and stored at −80 °C until untargeted plasma metabolomics was performed. Ultrahigh-performance liquid chromatography-tandem mass spectroscopy global metabolomics platform (UPLC-MS/MS) was performed at the laboratory of Metabolon, Inc. (Morrisville, NC). The study samples were randomized according to variables such as blood collection site, diet group, and race. Samples collected at different time points (baseline, 6 months, and 2 years) from the same individual were included in the same run adjacent to each other to minimize batch-varying effects. Quality control (QC) samples were evenly spaced among the injections. QC procedures included a pooled matrix sample generated by combining small volumes of each experimental sample (or alternatively a pool of well-characterized human plasma) used as a technical replicate throughout the dataset; extracted water samples as process blanks; and internal standards spiked into all samples to monitor instrument performance and aid chromatographic alignment. After metabolomic profiling, the dataset comprised 1697 biochemicals, including 1336 compounds of known identity (named biochemicals) and 361 compounds of unknown structural identity (unnamed biochemicals).

In the present analysis, erythritol (HMDB0002994) was the primary metabolite of interest; there was no missing value among the study samples. Considering the FDA approval of erythritol as a food additive in 2001 (GRAS Notice No. 76) and the timing of the trial (between 2004 and 2007), plasma erythritol levels in the present analysis are influenced by both endogenous production from glucose and exogenous dietary intake from natural and artificial sources. Mannitol/sorbitol (HMDB0000765, HMDB0000247) and arabitol/xylitol (L-Arabitol [HMDB0001851], D-Arabitol [HMDB0000568], D-Xylitol [HMDB0002917]) were examined for comparisons but were not chromatographically separated. Other artificial sweeteners (saccharin [HMDB0029723], sucralose [HMDB0031554], and acesulfame [HMDB0033585]) were analyzed only for baseline levels due to a high number of missing values that did not meet detection ranges.

### Measurements of glucose and insulin concentrations

The measurements of common clinical biomarkers were performed at the Pennington Biomedical Research Center laboratory. Fasting glucose was measured using a glucose oxidase electrode on a Beckman Coulter DXC 600 Pro (Brea, CA), and insulin was measured using an immunoassay with chemiluminescent detection on the Immulite 2000 analyzer (Diagnostic Products Corporation, Los Angeles, CA). Insulin resistance was assessed by the HOMA-IR based on values of fasting glucose and insulin [18].

### Assessments of covariates

Weight and waist circumference were measured at baseline, and every 6 months thereafter. Body mass index (BMI) (kg/m²) was calculated as weight in kilograms divided by height in meters squared. We analyzed habitual total physical activity levels at baseline and 6 months, using the Baecke questionnaires [19]. Details on assessments of habitual physical activity were described previously [20]. Twenty-four-hour urine samples were obtained repeatedly, and changes in the urine albumin-to-creatinine ratio (ACR) were calculated as previously described [17]. Serum creatinine concentrations were measured only at pre-intervention and were available in a subsample of participants (*n* = 395); the estimated glomerular filtration rate (eGFR) was calculated [21].

### Statistical analysis

The primary outcomes were changes in insulin sensitivity (fasting insulin and HOMA-IR) and glycemic markers (fasting glucose and HbA1c) at 6 months and 2 years. Data on metabolites, insulin, and HOMA-IR were log-transformed to improve data normality. We first analyzed baseline associations of plasma erythritol, arabitol/xylitol, mannitol/sorbitol, and low-calorie sweetener metabolites as comparisons, with insulin resistance and glycemic markers. General linear models were used to estimate β and 95% CIs per 1 z-score (mean = 0, SD = 1) increment in each metabolite at baseline for the respective outcome after adjustment for covariates (age, sex, race, study site, and family history of diabetes) with either BMI or waist circumference. Outcome variables were z-score standardized to compare effect estimates across measures. Crude *p* values and false discovery rate-adjusted *p* values (*P*_*FDR*_) were calculated.

Changes (Δ) in log-transformed metabolites from baseline to 6 months were calculated; the general linear models were used to estimate effect size β (SE) per 1 SD decrease in each metabolite in response to the interventions for the respective outcome after adjusting for baseline levels of the respective outcome, baseline levels of the respective metabolite, and other covariates. As reported previously [22], the weight-loss diet interventions led to decreases in other circulating metabolites associated with T2D risk. We therefore estimated β per 1 z-score decrease (i.e., per –1 SD change) for the respective outcome. We also performed additional models adjusted for other covariates and for baseline and changes in body weight or waist circumference to examine whether associations were independent of smoking habits, baseline and changes in physical activity (tertiles or a missing indicator), urinary ACR (tertiles or a missing indicator), and adiposity. Of the 664 participants included in the analysis of erythritol changes, 96 and 85% had data on concurrent changes in physical activity index and urine ACR, respectively. Two participants with missing baseline ACR values were imputed with median. To assess clinical relevance, we also calculated percent changes in HOMA-IR and fasting insulin. In addition, associations of the initial (6-month) metabolite changes with long-term (2-year) improvements in the primary outcomes were examined. Trajectories of 2-year changes in insulin sensitivity were compared across the tertiles of erythritol changes; mixed-effects models were used to test the significance of time-varying associations. Sensitivity analysis was performed to test potential interactions between changes in metabolites and diet groups (low vs. high fat/carbohydrate intakes and carbohydrate amount [35, 45, 55, 65%E]) for the outcomes. Statistical analyses were performed with SAS version 9.4 (SAS Institute. Statistical tests were two-sided, and the *p* value < 0.05 was used as a statistically significant threshold, considering the correlations among the metabolites and outcomes.

## Results

Table 1 shows characteristics of study participants by tertiles of baseline plasma erythritol levels. Compared with participants in the lowest tertile, those in higher tertiles were more likely to be older, female, and White, and had higher levels of waist circumference, insulin resistance, fasting glucose, and serum creatinine, as well as lower eGFR. There were no significant differences in smoking, physical activity, dietary intakes of total energy, carbohydrates, or fat by plasma erythritol levels.

**Table 1:** Baseline characteristics of study participants according to the tertiles (T) of plasma erythritol levels.

Characteristic	Plasma erythritol			*P* value
	T1	T2	T3	
*N*	267	268	267	–
Age, y	47.9 (9.2)	50.4 (8.4)	54.4 (8.9)	<0.001
Women, %	191 [71.5]	168 [62.7]	150 [56.2]	0.001
White, %	178 [66.7]	221 [82.5]	237 [88.8]	<0.001
Family history of diabetes, %	87 [32.6]	86 [32.1]	68 [25.5]	0.134
Smoking habit				0.172
Never	173 [64.8]	156 [58.2]	148 [55.4]	–
Former	83 [31.1]	104 [38.8]	107 [40.1]	–
Current	11 [4.1]	8 [3.0]	12 [4.5]	–
Total physical activity index	7.1 (1.4)	7.0 (1.4)	7.0 (1.4)	0.477
High-fat diet group	126 [47.2]	132 [49.3]	145 [54.3]	0.239
High-protein diet group	133 [49.8]	127 [47.4]	137 [51.3]	0.657
Body mass index, kg/m^2^	32.5 (3.8)	32.7 (3.9)	32.9 (3.8)	0.209
Waist circumference, cm	101 (12)	103 (13)	106 (13)	<0.001
Glucose, mg/dl	90 (10)	93 (18)	94 (12)	<0.001
HbA1c, %	5.4 (0.4)	5.4 (0.5)	5.4 (0.4)	0.028
Log-transformed HOMA-IR	0.7 (0.6)	0.8 (0.6)	1.0 (0.6)	<0.001
Log-transformed fasting insulin	2.3 (0.6)	2.3 (0.6)	2.4 (0.5)	<0.001
Serum creatinine, mg/dl^a^	0.9 (0.2)	0.9 (0.2)	1.0 (0.2)	<0.001
Estimated glomerular filtration rate, ml/min/1.73 m²^a^	90.1 (15.2)	84.5 (14.5)	77.9 (14.4)	<0.001
Albumin-to-creatinine ratio, mg/g	6.8 (8.9)	5.6 (5.3)	6.1 (6.9)	0.274
Dietary intake from food records^b^				
Energy (E), kcal/day	1955 (618)	2026 (569)	1924 (513)	0.650
Cholesterol, mg/day	302 (126)	317 (138)	277 (116)	0.106
Carbohydrate, %E	44.6 (7.7)	44.4 (7.3)	45.5 (7.8)	0.344
Fat, %E	36.7 (6.1)	37.2 (5.7)	37.0 (6.1)	0.691
Protein, %E	18.5 (3.8)	17.9 (3)	17.7 (3.1)	0.039
Carbohydrate, g/day	218 (78)	223 (69)	219 (70)	0.877
Fat, g/day	81 (31)	85 (29)	79 (25)	0.636
Protein, g/day	88 (26)	90 (26)	84 (25)	0.223

Data are mean (SD) or *n* [%]. *P* value for trend for continuous variables, or *P* value by *χ*^2^ test for categorical variables.

^a^Food records were collected in 50% of the original study participants: *n* = 138 in T1, *n* = 140 in T2, and *n* = 134 in T3.

^b^Serum creatinine and estimated glomerular filtration rate were available in 395 participants: *n* = 130 in T1, *n* = 127 in T2, and *n* = 138 in T3.

**Fig. 1:**
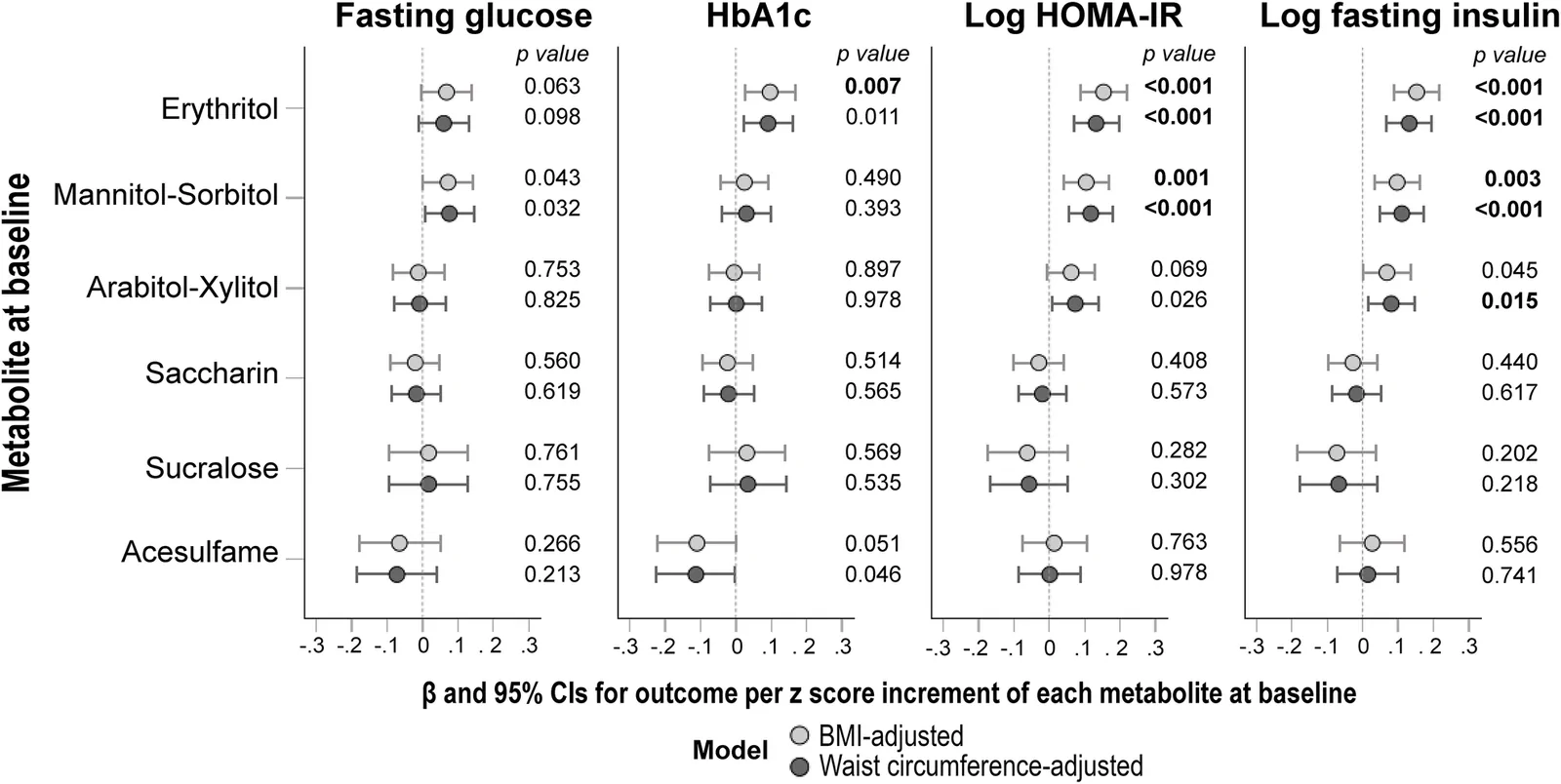
Associations of plasma erythritol, other sugar alcohols, and non-nutritive sweeter metabolites with baseline glycemic markers and insulin resistance. All outcomes and metabolites were z-score standardized (mean = 0 and SD = 1) before performing general linear models. Effect estimates (β) and 95% CIs per 1-SD increment for the respective outcome were estimated using models adjusting for age, sex, race, study site, family history of diabetes, and body mass index (BMI) (BMI-adjusted model: light gray circles) or waist circumference (waist circumference-adjusted model: dark gray circles). Bold *p* values indicate significant associations after false discovery rate (FDR) correction.

Higher levels of plasma erythritol were significantly associated with higher levels of HbA1c, log-transformed values of HOMA-IR, and fasting insulin at baseline (*p* < 0.001; *P*_FDR_ < 0.05) in models adjusting for demographic factors and general (assessed by BMI) or central adiposity (by waist circumference) (Fig. 1). Similar associations were observed between plasma mannitol/sorbitol and differences in HOMA-IR and fasting insulin at baseline (*P*_FDR_ < 0.05). Higher levels of arabitol/xylitol were related to higher levels of HOMA-IR (*p* = 0.026) and fasting insulin (*p* = 0.015) in models adjusting for waist circumference; however, the associations were not as robust as those observed for erythritol or mannitol/sorbitol. We found no significant association between the other artificial non-nutritive sweeteners (saccharin, sucralose, or acesulfame) and baseline glycemic markers or insulin resistance.

At 6 months in response to the interventions, the participants showed a large variability in changes (Δ) in plasma erythritol, arabitol/xylitol, and mannitol/sorbitol (Supplementary Fig. 1). There were significant positive correlations among changes in these metabolites, with a Pearson correlation coefficient [*r*] = 0.45 between Δerythritol and Δarabitol/xylitol (*p* < 0.001), and *r* = 0.10 between Δerythritol and Δmannitol/sorbitol (*p* = 0.007) (Supplementary Fig. 2). Overall, baseline characteristics (such as baseline smoking habit, physical activity, adiposity, glycemic/IR or kidney markers) were not significantly associated with Δerythritol in response to the interventions, except that participants who experienced declines in erythritol were more likely to be younger than those with increases in erythritol (Supplementary Table 1). We found that erythritol levels tended to decrease more in participants who consumed the lowest-carbohydrate (35%E) diet than in those who consumed the highest-carbohydrate diet (65%E) (Supplementary Fig. 3).

We then analyzed changes in glycemic markers and insulin sensitivity at 6 months and 2 years per 1 z-score reduction (i.e., –1 SD) of plasma erythritol and other polyols using models adjusted for diet group, age, demographic factors, BMI, baseline levels of the respective outcome, and baseline levels of each metabolite (Table 2). A greater decline in plasma erythritol was associated with larger reductions in log-transformed HOMA-IR (β –0.09 [SE 0.02]; *p* < 0.001) and log-transformed fasting insulin (β –0.09 [SE 0.02]; *p* < 0.001) at 6 months. When expressed as percent change, these estimates corresponded to changes of –7.9% (SE 2.2%; *p* < 0.001) in HOMA-IR and –7.4% (SE 2.0%; *p* < 0.001) in fasting insulin levels from baseline to 6 months. Similarly, greater declines in plasma arabitol/xylitol were related to more improvements in log-transformed HOMA-IR (β –0.06 [SE 0.02]; *p* = 0.002) and log-transformed fasting insulin (β –0.06 [SE 0.02]; *p* = 0.001) at 6 months. These associations remained significant in sensitivity models after further adjustment for smoking habits, physical activity, and baseline and concurrent changes in urine ACR, as well as baseline and concurrent changes in body weight or waist circumference (Supplementary Table 2).

**Table 2:** Changes (Δ) in glycemic markers and insulin sensitivity at 6 months and 2 years per 1 SD decline in plasma erythritol, arabitol/xylitol, or mannitol/sorbitol levels in response to interventions.

Follow-up time ΔOutcome	Mean of outcome	ΔErythritol			ΔArabitol-Xylitol			ΔMannitol-Sorbitol		
		N	β (SE)	*P*	*N*	β (SE)	*P*	*N*	β (SE)	*P*
At 6 months										
ΔFasting glucose	–2.43	664	–0.43 (0.35)	0.223	669	–0.40 (0.34)	0.237	668	0.19 (0.36)	0.590
ΔHbA1c	–0.08	663	0 (0.01)	0.997	668	–0.01 (0.01)	0.451	667	0.04 (0.01)	0.009
ΔLog-transformed HOMA-IR	–0.27	638	–0.09 (0.02)	<0.001	643	–0.06 (0.02)	0.002	642	0 (0.02)	0.945
ΔLog-transformed fasting insulin	–0.25	638	–0.09 (0.02)	<0.001	643	–0.06 (0.02)	0.001	642	0 (0.02)	0.879
At 2 years										
ΔFasting glucose	2.07	528	–1.0 (0.44)	0.025	532	–0.05 (0.42)	0.899	531	0 (0.46)	0.999
ΔHbA1c	–0.04	527	–0.05 (0.02)	0.019	531	0 (0.02)	0.859	530	0 (0.02)	0.955
ΔLog-transformed HOMA-IR	–0.12	512	–0.07 (0.02)	0.008	516	–0.02 (0.02)	0.478	515	0 (0.03)	0.929
ΔLog-transformed fasting insulin	–0.15	513	–0.06 (0.02)	0.010	517	–0.01 (0.02)	0.501	516	0 (0.02)	0.975

β (SE) represents changes in the respective outcome per z-score reduction (per –1 SD) of each metabolite from baseline to 6 months. Data were adjusted for age, sex, race, study site, diet group, family history of diabetes, body mass index, baseline value of the respective outcome, and baseline values of each metabolite.

In addition, the initial (6-month) declines in plasma erythritol were significantly predictive of the long-term (2-year) decreases in insulin sensitivity and glycemic markers (Table 2). Every 1 SD reduction of plasma erythritol during the initial 6 months was related to long-term (2-year) decreases in log-transformed HOMA-IR (β –0.07 [0.02], *p* = 0.008) and glycemic markers (β –1.0 [SE 0.44] mg/dL, *p* = 0.025 for fasting glucose; β –0.05% [SE 0.02%], *p* = 0.019 for HbA1c). On the other hand, we found no associations between Δarabitol/xylitol or Δmannitol/sorbitol with long-term improvements in glucose metabolism and insulin sensitivity at 2 years.

**Fig. 2:**
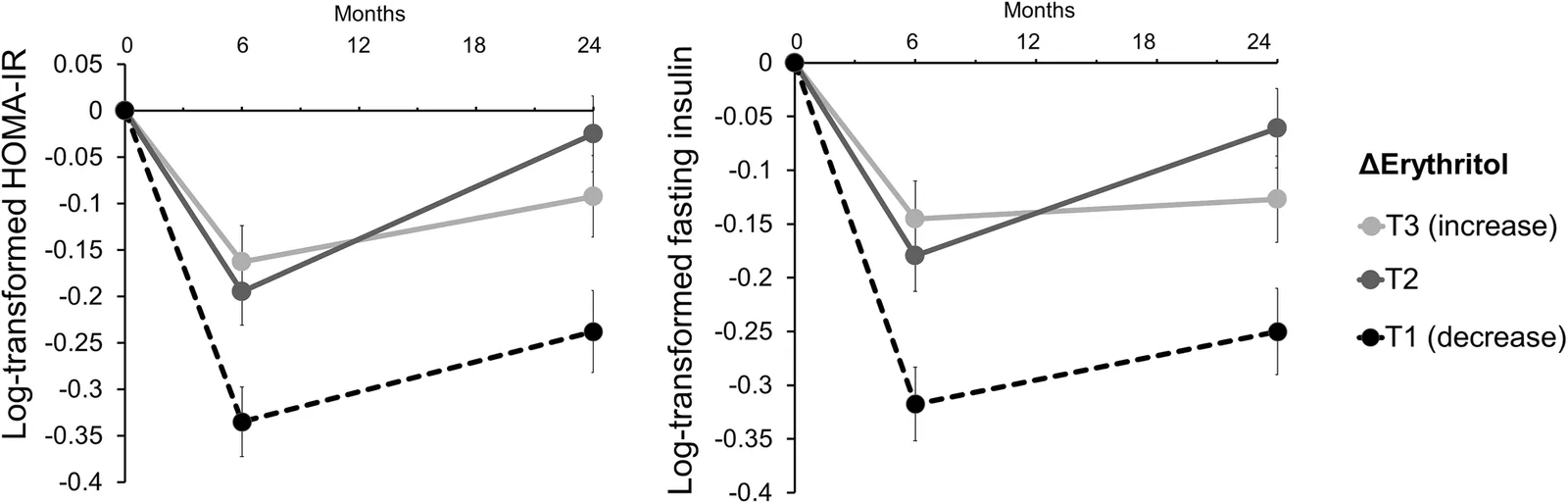
Trajectories of changes in HOMA-IR and fasting insulin levels over 2 years according to the tertile (T) categories of initial (6-month) changes in plasma erythritol. The lowest tertile (T1) category includes participants with the largest decreases in erythritol from baseline to 6 months after the interventions. Data are mean (SE) values after adjusting for age, sex, race, study site, family history of diabetes, diet group, body mass index, baseline value of erythritol, and baseline value of the respective outcome.

Figure 2 shows trajectories of changes in fasting insulin and HOMA-IR over 2 years according to the tertile (T) categories of the initial changes in plasma erythritol. People in the lowest tertile (T1) had decreases in erythritol, whereas those in higher tertiles (T2-T3) had increases or smaller reductions in erythritol based on the data distribution (Supplementary Fig. 1). Compared with participants in higher tertiles, those in the T1 with the largest decreases in erythritol showed consistently improved insulin sensitivity over 2 years, with no significant time-varying effects in the mixed models (*p* > 0.05). The T1 group showed clinically meaningful improvements in HOMA-IR from baseline, with adjusted mean percent changes of −16.4% (SE 3.8%) at 6 months and −10.9% (SE 4.8%) at 24 months. When we performed a secondary analysis to investigate associations considering dietary carbohydrate and fat intakes, we found significant interactions between Δarabitol/xylitol and dietary carbohydrate/fat intakes for changes in fasting insulin and HOMA-IR in both 6 months and 2 years (*P*_interaction_ < 0.05 for all) (Supplementary Tables 3 and 4). Also, the associations of Δarabitol/xylitol and outcomes were particularly significant among participants who consumed the lowest carbohydrate amount (35%E) during the interventions (*P*_interaction_ < 0.05). Similarly, significant interaction associations were observed between the tertiles of Δerythritol and dietary carbohydrates on 6-month and 2-year changes in HOMA-IR and fasting insulin (*P*_interaction_ < 0.05 for all). Among individuals who consumed the low-carbohydrate (35–45%E), high-fat (40%E) diet, smaller decreases in erythritol levels were related to smaller reductions in HOMA-IR and fasting insulin levels during 2 years (Supplementary Figs 4 and 5).

## Discussion

The present study showed that greater declines in plasma erythritol levels in response to weight-loss diet interventions were significantly associated with improved insulin sensitivity among adults with overweight or obesity. Similar associations were found for arabitol/xylitol. The initial reductions in plasma erythritol levels were significantly associated with long-term improvements in insulin sensitivity and glycemic markers over 2 years.

Erythritol synthesis may represent an adaptive response to oxidative stress associated with obesity and elevated blood glucose [23], and higher plasma sugar alcohol levels may be a risk factor for T2D among patients with obesity in this study. A cross-sectional study of women showed that higher plasma erythritol levels were associated with T2D and impaired fasting glucose [24]. Circulating erythritol was associated with T2D risk in an independent prospective cohort study [3]. In a nested population-based case-control study analyzing metabolomic pathways, signatures of dysregulation in the pentose phosphate pathway and elevated plasma erythritol were associated with diabetic retinopathy [14]. We recently found significant associations between high plasma erythritol levels and greater adiposity and diabetes-related markers in women from the Nurses’ Health Study and Women’s Lifestyle Validation Study [2], suggesting unfavorable associations between plasma erythritol levels and glycometabolic abnormalities [2]. Similarly, in the present study, the associations between plasma erythritol or mannitol/sorbitol and insulin resistance remained significant after adjustment for overall and central adiposity, suggesting that dysregulation of these sugar alcohols was closely related to insulin resistance in individuals with overweight or obesity.

Our study is novel in its analysis of temporal changes in erythritol and other polyols, and suggests that declines in plasma erythritol levels during the initial 6 months are associated with improved insulin sensitivity and glycemic markers at 2 years. We observed that participants who had the largest declines in erythritol levels (in the T1 group) in response to the interventions had clinically meaningful improvements in insulin sensitivity (an average improvement of 10% or greater) over 2 years. Associations of circulating erythritol levels with central adiposity and hyperglycemia may be regulated by pathways related to the endogenous production of erythritol [1]. Interestingly, we found that carbohydrate amount, in particular, lower carbohydrate intake, was significantly associated with greater reductions in plasma erythritol levels over 6 months. Erythritol is naturally present in certain foods, and elevated plasma erythritol levels can be observed in people who were not exposed to high levels of artificial erythritol as a food additive, as reported in our previous study [2]. When we explored potential factors associated with changes in plasma erythritol, baseline characteristics were not significant drivers, except that participants with declines in erythritol tended to be younger. This is consistent with a mouse study showing that plasma erythritol levels after a high-fat diet supplemented with erythritol were approximately twofold higher in older than younger mice on the same diet [12]. In a previous study of mice with high-fat diet-induced obesity (60% fat), supplementation with a non-nutritive sweetener (99% erythritol, 1% aspartame) in drinking water (4% for 4 weeks) affected glucose tolerance and was accompanied by hyperinsulinemia, compared with controls drinking normal water [25]. However, it remains unclear whether associations were independent of aspartame and high dietary fat intake. A study reported higher plasma erythritol levels in young mice fed a high-fat diet (45%) than in those fed a low-fat diet (16%), suggesting potential differences in endogenous production driven by dietary fat or other factors [12]. One study has found that consumption of 30% sucrose in water elevated plasma and urinary erythritol concentrations in both low-fat- and high-fat diet-fed mice [13], suggesting that carbohydrate quality may also be a contributing factor in erythritol metabolism. Our study could not determine the extent to which endogenous or exogenous factors contribute to variability in plasma erythritol changes in response to the interventions. We could speculate that dysregulation of the pentose phosphate pathway, which is associated with carbohydrate-rich diets or impaired glucose metabolism, might be improved during the interventions, and that such metabolic changes might be reflected in reductions in circulating erythritol levels.

We also observed that dietary carbohydrate and fat intakes significantly modified the associations between arabitol/xylitol (and the tertiles of erythritol change) and improvements in insulin sensitivity. Xylitol is a five-carbon polyol, naturally present in fruits, berries, and vegetables at very low concentrations, and a small percentage is also produced by the human body [26, 27]. Several acute interventional studies showed increases in plasma xylitol and small increases in blood glucose and insulin in response to various doses of xylitol [28–30]. High-fat diets typically include low carbohydrate content; it was difficult to determine which macronutrient primarily contributed to the associations. Nonetheless, our study underscores the importance of macronutrient composition in the relationship between changes in certain sugar alcohols, such as arabitol/xylitol and erythritol, and improvements in insulin sensitivity and glycemic status. Erythritol, sorbitol, and mannitol may not affect gut microbiota composition, although moderate doses of certain polyols could induce shifts in the gut microbiome in healthy people [31]. Xylitol may shift the gut microbiome [31], and erythritol solutions have also been linked to stimulating the release of gut hormones in a dose-dependent manner [32]. On the other hand, findings from recent Mendelian randomization studies examining potential causal associations between erythritol and cardiometabolic diseases are inconsistent [33, 34]; one study found no evidence supporting harmful cardiometabolic effects of erythritol [34]. It has also been reported that polyols show promise as alternative sweeteners and do not trigger acute or short-term impairments in post-load glycemic responses in studies, mainly those conducted in small numbers of healthy adults [15]. Further studies are warranted to examine the causality and biological mechanisms. In addition, the detected levels of polyols may not necessarily indicate an exogenous (dietary) source. Our study underscores the need for further research to clarify the contributions of exogenous vs. endogenous sources. However, feeding interventions alone may not fully resolve this distinction, highlighting the need for complementary approaches such as metabolic tracing to better understand polyol origins and dynamics in relation to T2D risk.

This study has several strengths. We repeatedly measured erythritol and other polyols to analyze dynamic changes in response to dietary interventions and showed associations between initial changes in these metabolites and long-term changes in glycemic measures and insulin sensitivity. Although we could not determine causality, the intervention design has advantages over observational studies in suggesting that dietary weight loss affects changes in these metabolites in the development and prevention of cardiometabolic abnormalities. Limitations were that we could not separately evaluate the impact of added (artificial) erythritol intake or changes in dietary erythritol intake. Further research is needed to clarify whether the association between circulating erythritol and improved insulin sensitivity is primarily due to endogenous production, dietary erythritol, or other artificial sweeteners used in combination. In addition, we could not determine whether changes in individual components of xylitol or sorbitol were related to the outcomes. It was challenging to identify an independent dietary intervention trial with repeated metabolite measurements comparable to the POUNDS Lost trial. Further investigations with repeated metabolomics data are warranted to validate the present study findings.

In conclusion, reductions in plasma erythritol were early indicators of long-term improvements in insulin sensitivity and glycemic status during weight-loss intervention. Plasma erythritol may be a pivotal factor in managing metabolic abnormalities in adults with overweight or obesity.

## Supplementary information

Online Supplementary Materials

## Data Availability

Datasets generated during and/or analyzed during the current study are not publicly available but are available upon reasonable request.
