# Supplementary material for: Declines in plasma erythritol levels and improvements in insulin sensitivity and glucose metabolism: POUNDS lost trial
Source: Int J Obes (Lond). 2026 Jul 21;50(10):2157–63. doi: 10.1038/s41366-026-02152-w (PMC13722594; doi:10.1038/s41366-026-02152-w)
Supplement: Supplementary file 1 [file 41366_2026_2152_MOESM1_ESM.pdf]

Supplementary Figure 1: Data distributions of 6-month changes in plasma erythritol, arabitol/xylitol, and mannitol/sorbitol

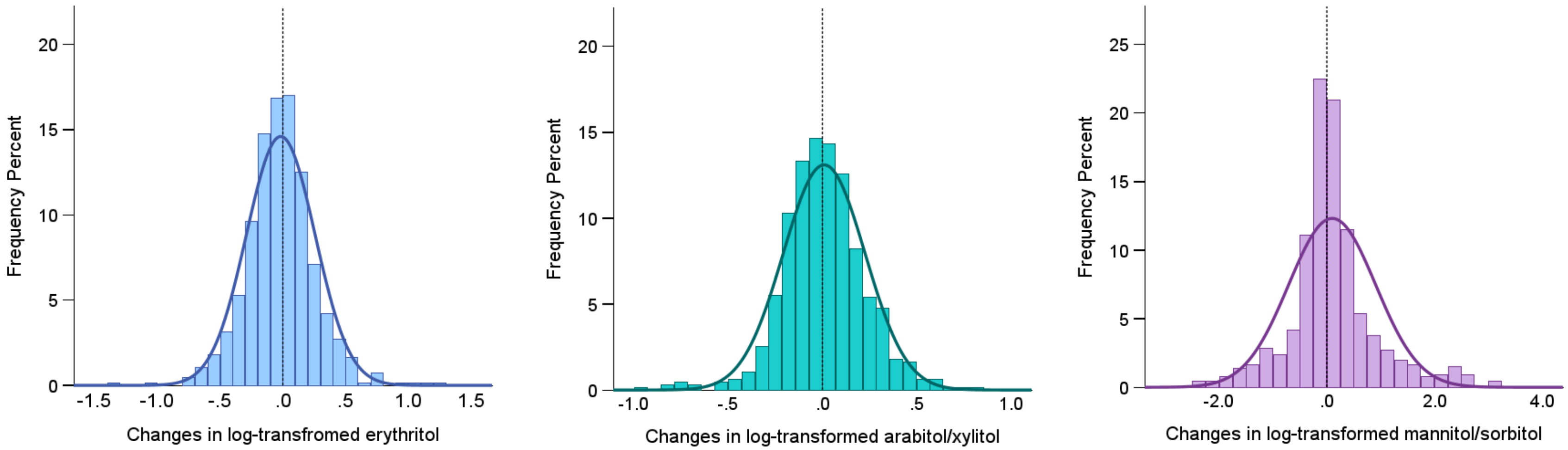

The dotted lines at 0 indicate no change in the respective metabolite from baseline to 6 months.

Supplementary Figure 2: Correlations among metabolites and metabolic factors.

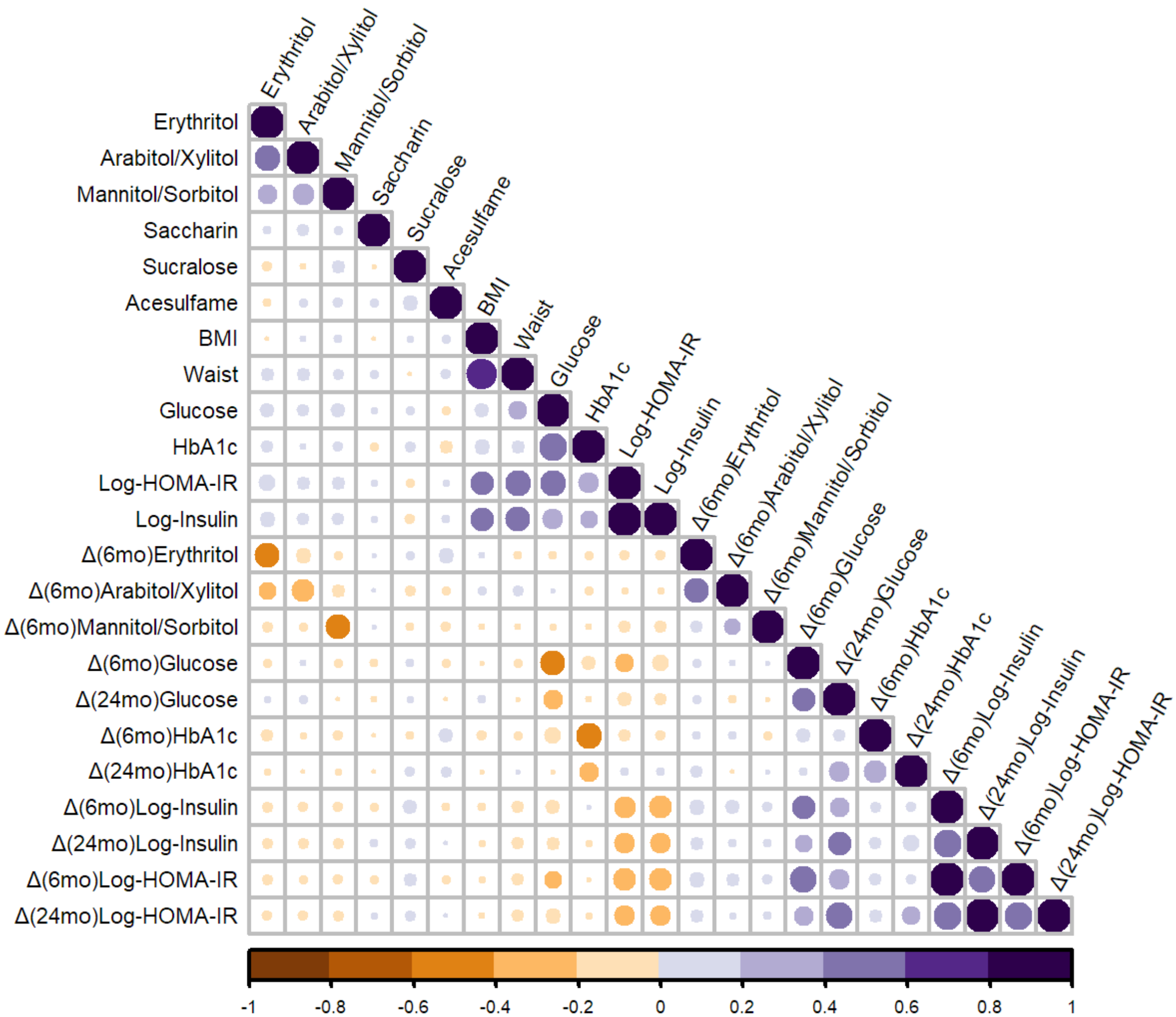

Supplementary Figure 3: Six-month changes in erythritol across diets with different carbohydrate content.

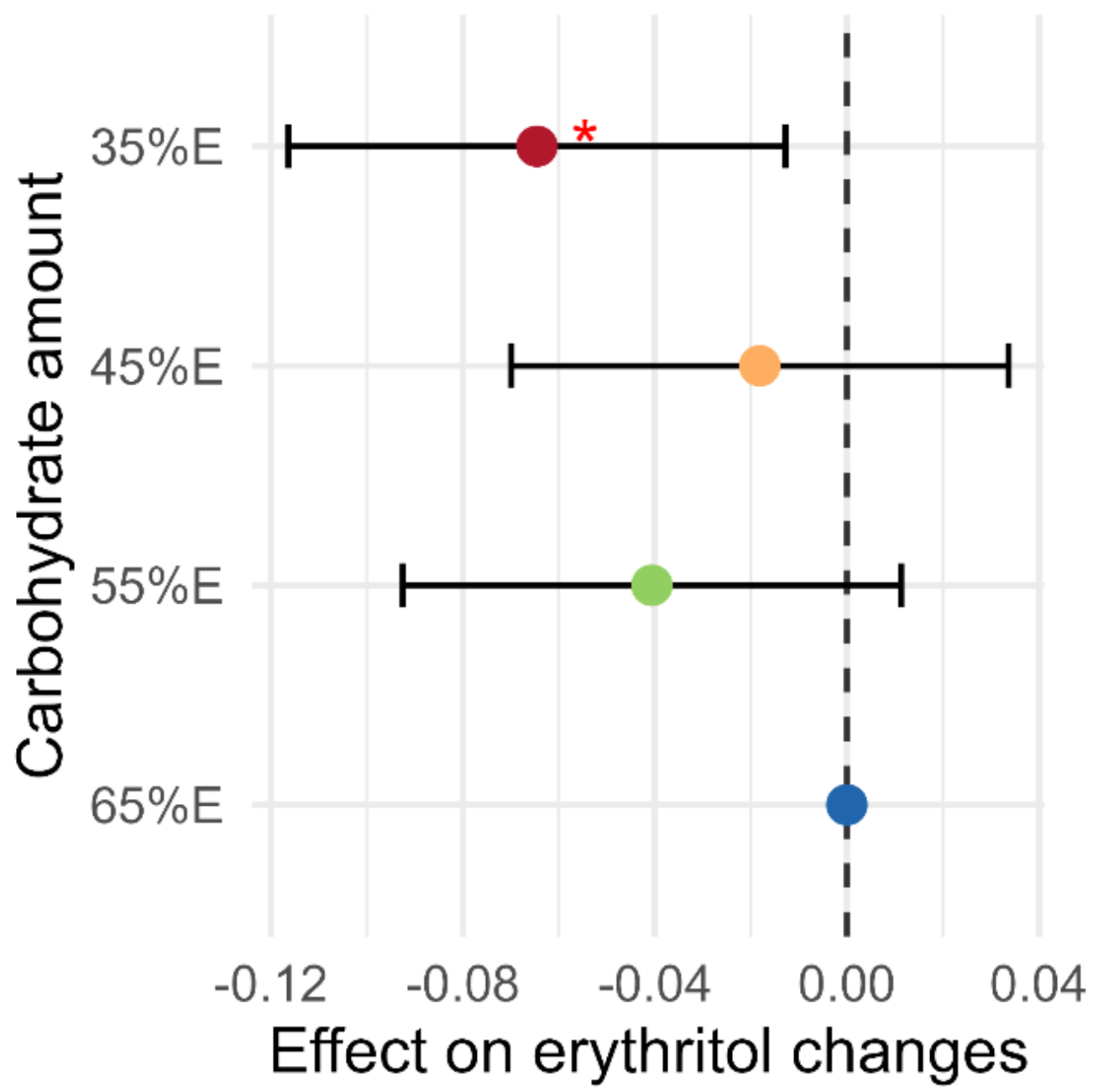

Data are β coefficients (95% CIs) for 6-month changes in plasma erythritol across diets with different carbohydrate content, with the highest carbohydrate group (65%E) as the reference. Data were adjusted for site, age, sex, race, and baseline BMI. **P* = 0.015 for the lowest carbohydrate diet group. P_{trend} =0.044.

Supplementary Figure 4: Trajectories of 2-year changes in HOMA-IR according to the tertile categories of changes in erythritol or arabitol/xylitol in the low-carb and high-fat diet group and the high-carb and low-fat diet group

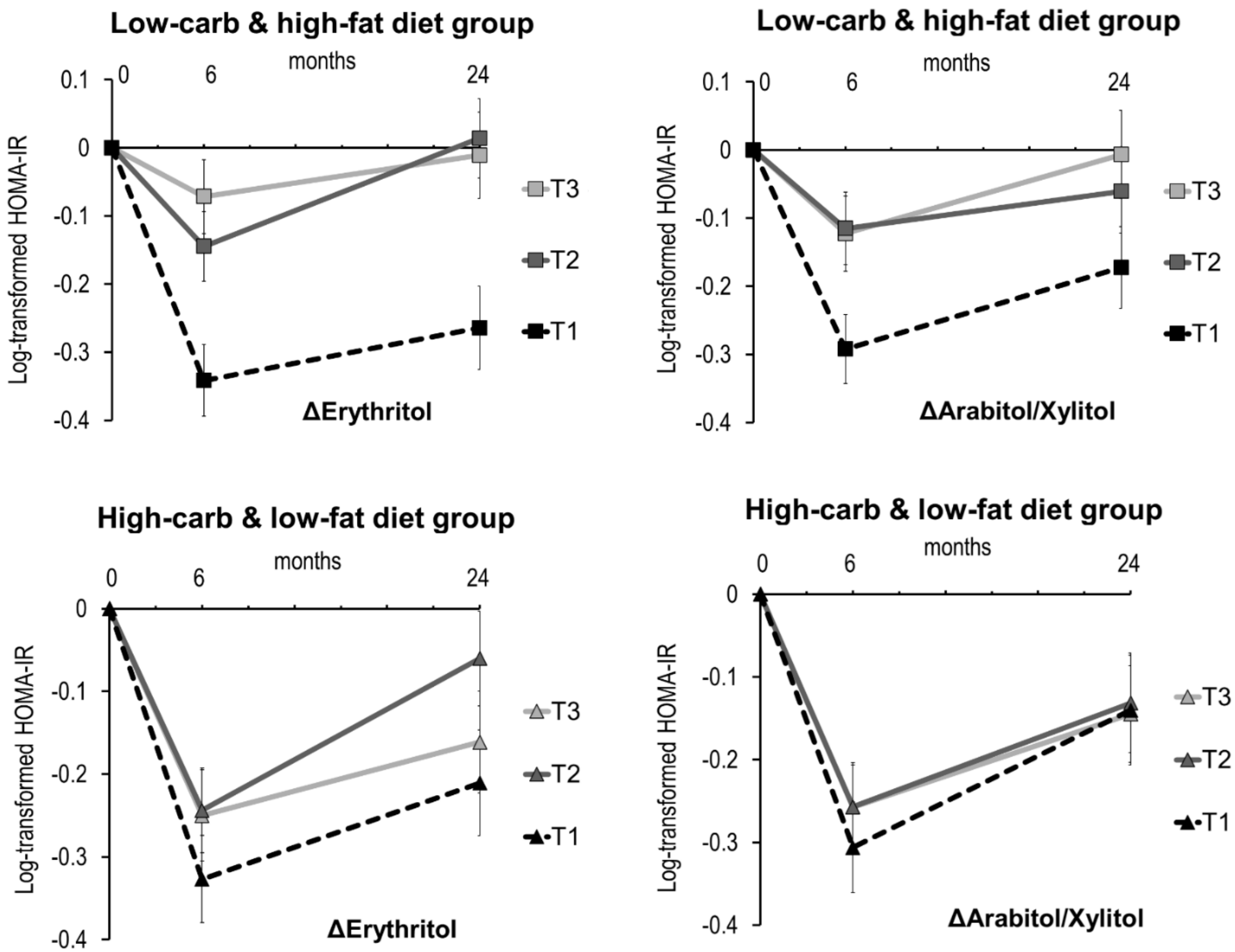

Supplementary Figure 5: Trajectories of 2-year changes in fasting insulin according to the tertile categories of changes in erythritol or arabitol/xylitol in the low-carb and high-fat diet group and the high-carb and low-fat diet group

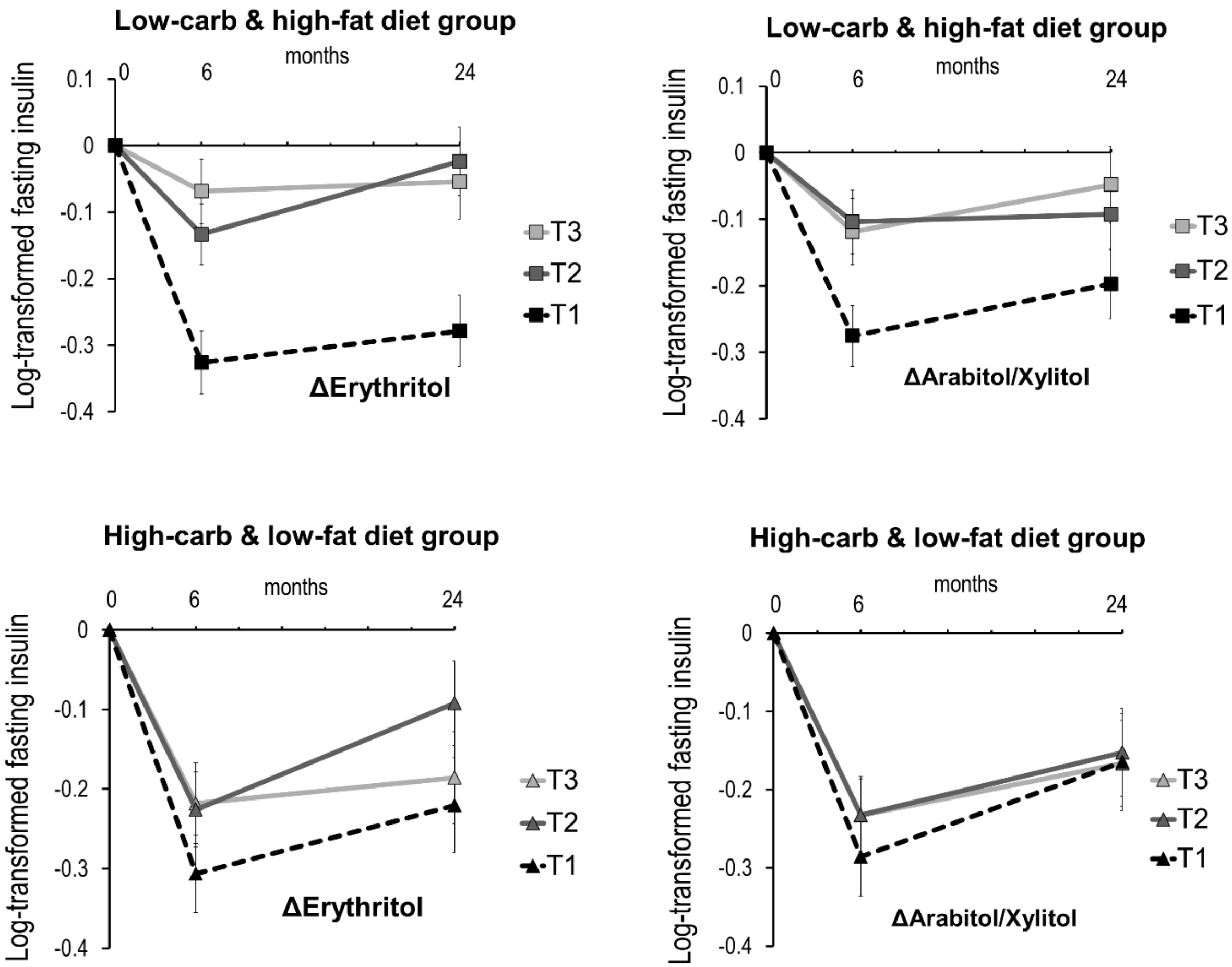

Supplementary Table 1: Comparison of characteristics according to 6-month changes in plasma erythritol.

	Changes in plasma erythritol			*p* value
Characteristic	T1	T2	T3	
N	221	222	221	-
Age, y	50.5 (0.7)	50 (0.6)	52.2 (0.7)	0.061
Women, %	133 [60.2]	132 [59.5]	132 [59.7]	0.988
White, %	187 [84.6]	177 [79.7]	182 [82.4]	0.404
Family history of diabetes	64 [29]	59 [26.6]	70 [31.7]	0.497
Smoking habit				0.519
Never	136 [61.5]	137 [61.7]	129 [58.4]	-
Former	81 [36.7]	76 [34.2]	82 [37.1]	-
Current	4 [1.8]	9 [4.1]	10 [4.5]	-
Total physical activity index*	7.2 (0.1)	6.9 (0.1)	7 (0.1)	0.195
Body mass index, kg/m^2	33 (0.3)	33.3 (0.3)	33.3 (0.3)	0.522
Waist circumference, cm	105.9 (0.8)	106.6 (0.8)	105.8 (0.9)	0.973
Glucose, mg/dl	92.3 (0.9)	93.2 (0.9)	92.7 (0.9)	0.764
HbA1c, %	5.5 (0.03)	5.5 (0.03)	5.5 (0.03)	0.556
Log-transformed HOMA-IR	0.89 (0.05)	1.01 (0.04)	0.94 (0.05)	0.457
Log-transformed fasting insulin	2.37 (0.04)	2.48 (0.04)	2.42 (0.04)	0.409
Serum creatinine, mg/dl†	1 (0.02)	1 (0.02)	1 (0.02)	0.556
Estimated glomerular filtration rate (eGFR), ml/min/1.73 m^2†	80.5 (1.4)	81.3 (1.4)	79.3 (1.6)	0.555
Urine albumin-creatinine ratio (ACR), mg/g	6.1 (0.6)	6.6 (0.6)	5.8 (0.6)	0.663

Data are presented as adjusted means (SE) for continuous variables or n (%) for categorical variables.

For continuous variables, means (SE) and p values were adjusted for age (except when age was analyzed), sex, race, study site, diet group, and baseline erythritol levels.

P values for trend were calculated for continuous variables, and p values were calculated using the χ^2 test for categorical variables.

*Total physical activity index was assessed using the Baecke questionnaire.

†Serum creatinine and eGFR were available in a subsample (n = 344).

Supplementary Table 2: Changes (Δ) in glycemic markers and insulin sensitivity at 6 months per 1 SD decrease in plasma erythritol, arabitol/xylitol, or mannitol/sorbitol levels in response to interventions in sensitivity models.

Model	ΔErythritol		ΔArabitol/Xylitol		ΔMannitol/Sorbitol	
ΔOutcome	β (SE)	*P*	β (SE)	*P*	β (SE)	*P*
**Model 1**						
ΔFasting glucose	-0.10 (0.35)	0.783	-0.23 (0.34)	0.491	0.23 (0.35)	0.514
ΔHbA1c	0.01 (0.01)	0.512	-0.01 (0.01)	0.560	0.04 (0.01)	0.011
ΔLog-transformed HOMA-IR	-0.04 (0.02)	0.018	-0.04 (0.02)	0.020	0 (0.02)	0.996
ΔLog-transformed fasting insulin	-0.04 (0.02)	0.014	-0.04 (0.02)	0.015	0 (0.02)	0.779
**Model 2**						
ΔFasting glucose	-0.14 (0.35)	0.695	-0.23 (0.34)	0.496	0.17 (0.35)	0.625
ΔHbA1c	0.01 (0.01)	0.579	-0.01 (0.01)	0.573	0.03 (0.01)	0.014
ΔLog-transformed HOMA-IR	-0.05 (0.02)	0.006	-0.04 (0.02)	0.018	-0.01 (0.02)	0.775
ΔLog-transformed fasting insulin	-0.05 (0.02)	0.004	-0.04 (0.02)	0.014	-0.01 (0.02)	0.573

Model 1 adjusted for age, sex, race, study site, diet group, smoking habit, family history of diabetes, baseline and concurrent (6-month) changes in total physical activity (PA) index, baseline and concurrent changes in urine albumin-creatinine ratio (ACR), baseline levels of the respective metabolite and outcome, initial body weight, and concurrent changes in body weight.

Model 2 adjusted for age, sex, race, study site, diet group, smoking habit, family history of diabetes, baseline and concurrent changes in total PA index, baseline and concurrent changes in urine ACR, baseline levels of the respective metabolite and outcome, initial waist circumference, and concurrent changes in waist circumference.

Supplementary Table 3: Changes (Δ) in glycemic markers and insulin sensitivity per 1 SD decrease in plasma arabitol/xylitol by dietary carbohydrate (carb) and fat intakes

**Follow-up time**	Low-carb (& high-fat) diet group			High-carb (& low-fat) diet group			*P* interaction
ΔOutcome	N	β (SE)	*P*	N	β (SE)	*P*	
**At 6 months**							
ΔFasting glucose	333	-0.94 (0.55)	0.09	336	0.04 (0.42)	0.92	0.048
ΔHbA1c	332	-0.02 (0.02)	0.3	336	0 (0.02)	0.92	0.56
ΔLog-transformed HOMA-IR	323	-0.122 (0.032)	0.0001	320	-0.022 (0.027)	0.42	0.024
ΔLog-transformed fasting insulin	323	-0.112 (0.029)	0.0001	320	-0.023 (0.025)	0.37	0.037
**At 2 years**							
ΔFasting glucose	263	-0.14 (0.72)	0.84	269	-0.01 (0.48)	0.99	0.29
ΔHbA1c	263	-0.03 (0.04)	0.46	268	0.03 (0.02)	0.07	0.037
ΔLog-transformed HOMA-IR	256	-0.062 (0.036)	0.08	260	0.007 (0.033)	0.83	0.042
ΔLog-transformed fasting insulin	257	-0.057 (0.032)	0.07	260	0.008 (0.031)	0.8	0.0498

β (SE) are changes in the respective outcome per z-score reduction (per –1 SD) of arabitol/xylitol changes (Δ) from baseline to 6 months. Data were adjusted for age, sex, race, study site, family history of diabetes, body mass index, baseline value of the respective outcome, and each metabolite at baseline. *P* interactions between Δarabitol/xylitol (continuous) and low-/high-carb diet groups (2 groups) for the respective outcome.

Supplementary Table 4: Changes (Δ) in glycemic markers and insulin sensitivity per 1 SD decrease in plasma arabitol/xylitol by dietary carbohydrate intakes (35-65%energy [%E]).

**Follow-up time**	35%E		45%E		55%E		65%E		P interaction
ΔOutcome	β (SE)	*P*	β (SE)	*P*	β (SE)	*P*	β (SE)	*P*	
**At 6 months**									
ΔFasting glucose	-1.5 (0.8)	0.051	-0.3 (0.8)	0.69	0.6 (0.6)	0.36	-0.3 (0.6)	0.64	0.036
ΔHbA1c	0 (0)	0.48	0 (0)	0.43	0 (0)	0.44	0 (0)	0.39	0.407
ΔLog-transformed HOMA-IR	-0.16 (0.05)	0.0004	-0.07 (0.05)	0.16	-0.01 (0.05)	0.78	-0.01 (0.04)	0.72	0.006
ΔLog-transformed fasting insulin	-0.14 (0.04)	0.0005	-0.06 (0.04)	0.14	-0.02 (0.04)	0.68	-0.01 (0.03)	0.79	0.009
**At 2 years**									
ΔFasting glucose	-0.1 (1)	0.92	-0.2 (1.1)	0.87	1.5 (0.7)	0.04	-0.7 (0.7)	0.3	0.6
ΔHbA1c	0 (0.1)	0.7	0 (0)	0.15	0 (0)	0.52	0 (0)	0.12	0.056
ΔLog-transformed HOMA-IR	-0.1 (0.05)	0.039	0 (0.06)	0.94	0 (0.05)	0.93	0.05 (0.05)	0.32	0.012
ΔLog-transformed fasting insulin	-0.1 (0.05)	0.036	0 (0.05)	>0.99	-0.02 (0.05)	0.64	0.05 (0.04)	0.21	0.01

β (SE) are changes in the respective outcome per z-score reduction (per –1 SD) of arabitol/xylitol changes (Δ) from baseline to 6 months. Data were adjusted for age, sex, race, study site, family history of diabetes, body mass index, baseline value of the respective outcome, and each metabolite at baseline. P interactions between Δarabitol/xylitol (continuous) and carbohydrate amount for the respective outcome.
